# Tackling obesity in mental health secure units: a mixed method synthesis of available evidence

**DOI:** 10.1192/bjo.2018.26

**Published:** 2018-07-24

**Authors:** Maxine Johnson, Matthew Day, Rajesh Moholkar, Paul Gilluley, Elizabeth Goyder

**Affiliations:** Research Fellow, School of Health and Related Research (ScHARR), University of Sheffield, Sheffield, UK; Consultant in Public Health for Specialised Commissioning, Public Health England, UK; Consultant Forensic Psychiatrist, Reaside Clinic, and Lecturer, School of Psychology, Birmingham University, Birmingham, UK; Consultant Forensic Psychiatrist and Head of Forensic Services, East London NHS Foundation Trust, London, UK; Professor of Public Health, School of Health and Related Research (ScHARR), University of Sheffield, Sheffield, UK

**Keywords:** Forensic mental health services, primary care, patients

## Abstract

**Background:**

The prevalence and incidence of obesity are high in people with severe mental illness (SMI). In England, around 6000 people with SMI access care from secure mental health units. There is currently no specific guidance on how to reduce the risk of obesity-related morbidity and mortality in this population.

**Aims:**

To identify international evidence that addresses the issue of obesity in mental health secure units.

**Method:**

A mixed method review of evidence (published 2000–2015) was carried out to assess obesity prevalence, intervention and policy change, as well as barriers to change.

**Results:**

Evidence from 22 mainly small, non-comparator studies (reported in 21 papers) using a range of methods was reviewed. Dietary, physical activity and cultural interventions being implemented within secure units to address the problem of obesity showed some promising outcomes for physical health and health education. These were facilitated by adequate organisational resources, staff training and motivated staff. Holistic interventions that included a social and/or competitive element were more likely to be taken up. Involving patients in decision-making mediated the tension between facilitating behaviour change and imposing control. Barriers to successful outcomes included patient movement in and out of units, severity of mental health condition and resistance to change by patients and staff.

**Conclusions:**

Despite the promising outcomes reported, further assessment is needed of the feasibility, acceptability and effectiveness of interventions and policies targeting the obesogenic environment, using robust research methods.

**Declaration of interest:**

None.

Obesity rates are higher in people with severe mental illness (SMI) than in the general population,^1^ owing to the effects of antipsychotic and antidepressant medications, diet and lack of adequate physical activity. For example, one review showed that around half of people with SMI fail to meet recommended levels of physical activity and many remain sedentary for up to 8 h per day.^2^ Patients with schizophrenia have a mortality rate 2–3 times greater than that of the general population, owing to conditions such as cardiovascular disease,^3^ which are associated with smoking and obesity.^4^ Reviews show that risk from metabolic syndrome is elevated compared with the general population (relative risk 1.58; 95% CI 1.35–1.86; *P* < 0.001), with no significant differences in risk between SMI diagnoses but significant variation across medication type,^5^ and one in ten individuals with SMI is diagnosed with type 2 diabetes.^6^ There is evidence that dietician-led nutritional interventions for individuals with SMI living in the community can influence weight gain.^7^

Current guidance recommends that all patients admitted to hospital receive body mass index (BMI) assessment on admission, along with interventions to motivate lifestyle change where necessary.^8^ Long *et al*^9^ reviewed evidence on promoting healthy lifestyles in mental health secure units, concluding that a change of culture is required that involves staff and patients. Currently, in England, around 6000 patients with severe mental health problems are detained within around 150 low, 65 medium and three high secure units,^10^ yet there is no specific guidance relating to intervention for managing weight or preventing obesity in these settings. A Care Quality Commission for Mental Health report^11^ highlighted the importance, identified in the National Health Service (NHS) England Five Year Forward View,^12^ of providing equitable physical healthcare for detained patients in order to limit mortality.

NHS England commissioned a review of existing evidence in this area, carried out and reported as part of a funded secondment for the researcher (M.J.) with Public Health England.^13^ In this paper we summarise the main findings of the review and discuss them relation to the broader literature.

## Aims

Along with a clinical reference group, we formulated a number of review aims. These were to identify the reported extent of obesity in low, medium and high secure mental health units; any interventions to tackle obesity or to manage weight that were being evaluated in these settings; and the acceptability and feasibility of interventions for stakeholders.

## Method

We carried out a mixed method review, which involved systematically reviewing and synthesising evidence obtained using different research methods about the same topic. This type of review is increasingly being carried out to guide decision-making.^14^ We followed the PRISMA guidelines, which are suitable for a range of review types.^15^ Initial scoping searches indicated that the body of published evidence relating specifically to mental health secure units would be limited, and that stakeholder input would support our task in identifying any new evidence and the feasibility of reported interventions. We therefore used a combination of mixed method review and stakeholder consultation methods to address the research questions.

A search strategy was developed with the assistance of a qualified information specialist (N.D.). An iterative search process was used with a range of MESH and free text terms to search MEDLINE, the Cochrane Library, PsychINFO, CINAHL, ASSIA and Social Science Abstracts for articles published between 2000 and 2015. We supplemented these searches using specific online resources such as Social Care Online and the Mental Health Foundation website, and with Google searches for grey literature (unpublished work). Reference list checking and citation searches were carried out based on retrieved articles. References were shared with the advisory team for feedback at regular intervals.

### Inclusion criteria

We included international studies carried out in Organisation for Economic Co-operation and Development (OECD) countries and published in English, as well as UK unpublished articles from 2000 to the present, using any study design that produced quantitative or qualitative outcomes. The relevant population was adults of any age (male and female) residing in mental health secure units (or their international equivalent), with any SMI diagnosis, as well as healthcare professionals providing care in those units. We were interested in epidemiological information, intervention/policy evaluation and views or survey data regarding the obesogenic environment in this specialist setting. We considered any non-pharmacological intervention compared with usual care.

### Data extraction and quality assessment

Citations were stored in reference manager software and screened for relevance to the review question by a researcher (M.J.). Those that met the inclusion criteria for population, setting, methods and topic (see above section) were tagged as such and retrieved as full papers. Full papers were submitted for further screening and discussion with a member of the team (M.D.) to ensure they met the inclusion criteria. Data were extracted from the final set of included studies using piloted extraction forms designed to include information from each type of study. Data extractions were carried out by one reviewer (M.J.), with 35% of the extractions being checked for accuracy by a second reviewer (D.C.).

Included papers were also assessed for quality using the Mixed Methods Appraisal Tool,^16^ which was specifically designed for critically assessing a body of mixed method studies or mixed method papers. The single included randomised controlled trial (RCT) was assessed using the Critical Appraisal Skills Programme tool for RCTs.^17^ Assessments were double checked by one reviewer (M.J.). As we aimed to provide comprehensive coverage of a relatively small body of evidence and were not seeking to compare effectiveness data, we did not exclude papers on the basis of quality assessment or score individual papers on quality. Instead, factors that did not fully meet the criteria used for quality assessment were noted for each study in order to identify the main issues arising from the studies. The main factors noted included lack of a comparator, and participant refusal/drop-out. These factors highlight the challenges facing researchers carrying out studies within mental health secure unit settings, for example, providing matched control groups and ensuring low attrition.

### Data synthesis

Extracted survey and quantitative data were categorised by study design, intervention type and population. Qualitative data were thematically analysed for mitigating or moderating factors relating to implementing interventions.

### Research Ethics

Ethical approval was not required for this study as data collection did not involve human participation.

## Results

### Study characteristics

**Fig. 1:**
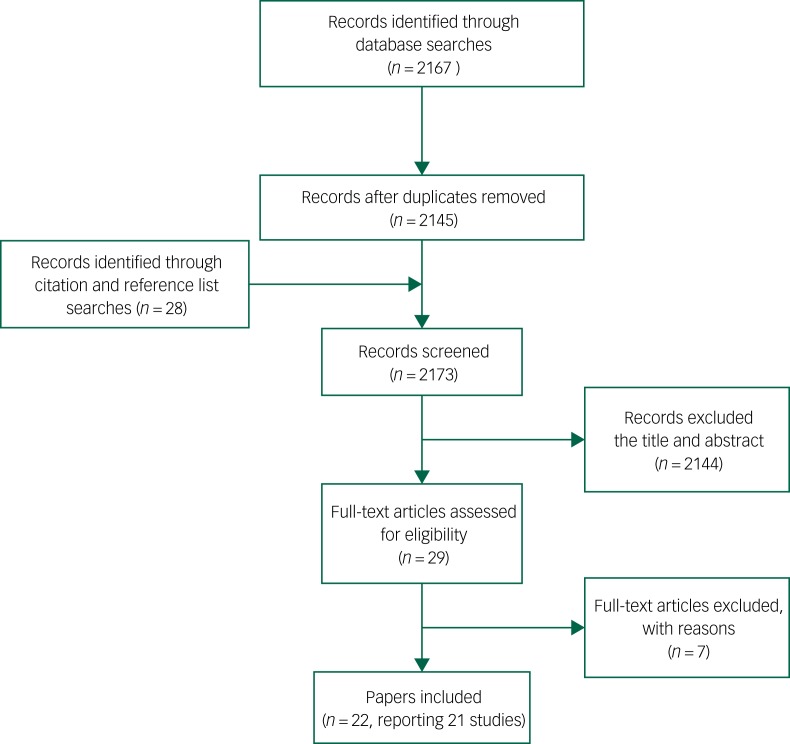
PRISMA diagram.

Following de-duplication, 2145 citations were sifted for relevance, and reference list checking and citation searches resulted in a further 28 articles. A total of 2144 papers were rejected at title/abstract level. Of the remaining 29 full papers, seven papers were rejected and 22 (describing 21 studies) were included (Fig. 1 and Table 1). No retrieved non-English language citation met all our inclusion criteria; therefore, we were reassured that no important citation was rejected.

**Table 1:** Summary of included papers^a^

Author/date	Focus of study	Study design/duration	Setting	Participants Sample size	Data collection methods
Bacon 2012^34^	Effect of Nintendo Wii Fit use on physical activity	Mixed methods Before-after 8 weeks	Australia Secure mental health hospital	Patients BMI 25–32 *n* = 2	Accelerometer readings Participant observation Interviews
Cormac 2005^18^	Health risk factors	Cross-sectional	UK High secure hospital	*N* = 248	Questionnaire Case notes
Cormac 2008^19^	Weight management/fitness service	Before-after 10–12 week programme	UK High secure psychiatric unit	Patients Completed *n* = 46	Physical and fitness measurements
Cormac 2013^20^ (follow-up to^19^		As above Findings after 10–12 sessions		As above 120 completed	
Faulkner 2002^21^	Nurses’ perceptions of the role of physical activity	Qualitative	UK mental health trust	In-patient MH nurses: *N* = 12	Interviews
Forsyth 2012^36^	Training needs of nursing staff (nutrition)	Qualitative	New Zealand Forensic rehabilitation	Nursing staff *N* = 11	Interviews Questionnaire
Harper 2008^22^	Expenditure/requisitions for foodstuffs by patients	Cross-sectional	UK High secure unit	Patients One unit	Summary data State benefit Food expenditure
Haw 2011a^23^	Proportion of overweight/obese patients	Cross-sectional	UK Secure psychiatric unit	Patients *N* = 234	Routine data
Haw 2011b^24^	In-patient weight management	Cross-sectional	UK Secure forensic units	Consultant psychiatrists *N* = 183 analysed	Questionnaire
Hjorth 2014^38^	Physical fitness programme	RCT 12 months	Denmark In-patient facilities (6)	Patients *N* = 85	Physiological measures Ratings
Long 2009^25^	Nutrition/eating habits	Cross-sectional Qualitative	UK Secure units (3)	Female patients *N* = 28	Questionnaire Observation
Long 2014^26^	Incidence of obesity and complications	Cross-sectional	UK Secure unit	*N* = 351	Routine data Risk screening Attendance data
Long 2015^27^	Effectiveness of interventions to increase motivation for PA participation	Before-After evaluation 3 months	UK Secure unit Low/medium secure wards	Female patients *N* = 32	Questionnaire
Kasmi 2009^28^	Number of takeaways delivered over 21 days	Cross-sectional	UK Medium secure unit	Patients	Survey
McCrarren 2013^39^ (Unpublished thesis)	Nutritional knowledge	Cross-sectional	Ireland Forensic mental health settings	Mental health nurses (*n* = 75)	Questionnaire
Meiklejohn 2003^29^	Physical healthcare needs	Cross-sectional	UK Medium secure unit	Patients *N* = 56	BMI measures Interviews
Oakley 2013^30^	Weight management strategies	Cross-sectional	UK Medium secure units (67)	Adult and adolescent patients	On-line survey
Prebble 2011^37^	Healthy living programmes	Case study	New Zealand forensic facilities (2)	HCPs (*n* = 17) Patients (*n* = 15)	Interviews Meetings Case notes
Rylance 2012^31^	How nurses perceive their role in physical care of patients	Qualitative	UK Acute in-patient unit	Mental health nurses *N* = 6	Semi-structured interviews
Savage 2009^32^	Initiative to increase engagement with PA	Before-after evaluation 12 weeks	UK Medium secure forensic unit (1)	Female patients *N* = 6	Personal training mood measure Views Attendance
Vasudev 2012^33^	Maintaining a physical health record sheet	Pilot evaluation 12 months	UK Medium secure unit (1)	Male patients *N* = 15	6 months audit of records
Wynaden 2012^35^	Healthy lifestyle programme	Mixed method evaluation 6 months	Australia State secure forensic mental health unit	Patients *N* = 56	Self-report questionnaire

a. Adapted from PHE 2016^13^ under the terms of the Open Government Licence v3.0 (https://www.nationalarchives.gov.uk/doc/open-government-licence/version/3/).

The majority of papers (16) were published in the UK,^18^^–^^33^ while the remaining papers were published in Australia,^34^^,^^35^ New Zealand,^36^^,^^37^ Denmark^38^ and Ireland (unpublished thesis).^39^

Three included papers focused on the prevalence of overweight and obesity within UK mental health secure units.^18^^,^^23^^,^^26^ Nine papers (describing eight studies) presented evaluations of individual-, group- or ward-level interventions.^19^^,^^20^^,^^27^^,^^32^^–^^35^^,^^37^^,^^38^ Of these, only one used a (cluster) RCT,^38^ with the remaining evaluations having no comparator. Two evaluations recruited only female patients,^27^^,^^32^ and one focused on males.^33^ Eight cross-sectional studies of health professionals or patients examine factors relating to the obesogenic environment, such as access to weight management strategies,^24^^,^^30^ the level of nutritional knowledge among staff,^39^ patient physical health needs,^29^ nutritional habits,^25^ spending on unhealthy food items^22^ and the ordering of takeaway meals.^28^

Four qualitative papers used interview methods to explore nurses’ perceptions of their role in promoting physical activity,^21^ giving nutritional advice^36^ and carrying out physical care,^31^ or about enablers to implementing healthy living programmes within the unit.^37^ Although included papers had a specific focus, the reported findings of evaluations and staff perceptions also provided additional information such as the extent of obesity and challenges to changing lifestyle behaviours in their particular setting, which are reported within the presented themes.

In terms of quality, only one study included a comparator and most cross-sectional studies had response rates of less than 60%. A number of studies could be described as ‘natural experiments’,^40^ where unit or ward changes are made and the differences to a range of outcomes over time assessed. Assessment in these cases was carried out using quantitative and qualitative methods, for example, observation of specific behaviours. As there is a recognised body of evidence regarding weight management interventions in the general population,^8^ the main aim of this review was to explore the strategies used and assessed within mental health secure units to address obesity in order to identify those that might show promise for future evaluation on a larger scale in these settings.

## Thematic synthesis

The literature provides mixed method evidence from mental health secure units across three main domains: the extent of the obesity issue and how it is monitored; what is being done to address obesity; and the challenges and facilitators that arise when addressing obesity in this setting. The following section provides a more detailed account of these overarching themes.

### Extent of the problem: obesity patterns in secure units

Studies identified a higher prevalence of obesity in UK secure settings than in the general population.^18^^,^^23^^,^^26^ The problem is exacerbated by the need for medication that increases the tendency for weight gain.^31^^,^^36^^,^^37^ Women present different obesity patterns to men across obesity levels I to III and across time.^18^^,^^23^^,^^26^ This could be due to challenges that women face in carrying out physical activity within the units,^26^ suggesting a need to cater for a range of capabilities when motivating patients and designing physical activities.

### Assessing and monitoring weight/physical health

The recent assertion that parity of esteem between physical and mental healthcare is needed^41^ points toward primary care-style assessment and monitoring of mental healthcare patients. Studies included in this review showed that monitoring the weight of patients as part of routine physical healthcare in secure units could be planned or opportunistic^24^^,^^31^ and dependent on available equipment and trained staff.^31^^,^^36^ Nurses working in the units reported that they did not feel sufficiently well trained to carry out physical healthcare,^31^ and lack of immediate access to primary care-trained nurses, general practitioners^29^ or dieticians^24^ was a reported barrier to maintaining physical healthcare.^33^ This evidence suggests that physical health promotion delivery can be suboptimal because the priority for busy mental health professionals has been patient safety, for example, reducing the risks posed by mental illness (including suicide)^21^ and improving mental health, rather than physical health outcomes.^31^ Indeed, in one qualitative study, a mental health nurse described physical health issues as ‘… *seem to be put to the back. You know, take a back burner*’^31^ (p. 17).

### Unit facilities

In a number of included papers, authors identified that lifestyle change is dependent on available facilities within units to support planned activities. For example, inadequate kitchen facilities, with small spaces and no freezer, restricted the ability to produce healthy meals on the ward.^37^ Increasing physical activity within the confined spaces of mental health secure units was also a reported issue,^21^^,^^37^ requiring imaginative development of space to include gymnasia, sport and recreational areas, and, where possible, a swimming pool.

Access to appropriate equipment and clothing is also necessary to encourage participation.^38^ One suggested way of increasing access was to provide opportunities to purchase reasonably priced sports clothing online or at the secure unit shop. This has a secondary effect on lifestyle by limiting overspending on high-calorie foods, which has been reported as a historical barrier to weight management.^22^

Physical activity programmes or classes were reported to be delivered by members of staff or qualified trainers, although staff shortages^20^^,^^24^ or the unwillingness of staff to perceive physical activity facilitation as part of their role^21^^,^^27^^,^^34^^,^^37^ were potential barriers to increasing physical activity within units. Encouraging gradual, small changes in staff attitudes or obtaining the support of qualified fitness trainers^29^^,^^33^ were suggested ways forward.

### Patient education and staff training

A number of areas were identified in the literature where education could play a part in improving patient lifestyle behaviours. Weight management groups were offered in one study so that patients could monitor their progress and learn general principles of lifestyle change, although these were poorly attended.^29^ Nutritional education was often incorporated into multi-component interventions that also addressed eating behaviours.^24^^,^^25^ Motivational support was important for maintenance, including advice on setting goals, recording achievements and accessing follow-up support in order to overcome challenges.^25^ As excessive disposable income was identified as a contributing factor to purchasing food of high calorific value, money management was a suggested topic for education.^22^ However, mental health nurses delivering lifestyle education were reported to potentially lack nutritional knowledge^37^^,^^39^ and confidence^21^^,^^31^ in delivering education and advice. This suggests a need for staff training and access to specialists such as dieticians and fitness trainers.

### Interventions for lifestyle behaviour change

Only one RCT (cluster) was identified from the searches. The trial, carried out in Denmark, assessed motivational and awareness-raising initiatives compared with usual care. Intervention sites showed a significant decrease in waist circumference of −3.1 cm (*P* = 0.018) at 12 months compared with controls, although it was possible that control sites were inadvertently contaminated through carrying out routine measurements that could have led to intervention.^38^

A range of physical health monitoring and lifestyle behaviour programmes were evaluated in small-scale, mainly in-house studies that lacked a comparator. In order to test the feasibility of recording physical health monitoring within one UK-based unit, audited completion of a monitoring sheet was evaluated. This resulted in 100% completion but no reduction in mean BMI at 12 months. Authors reported that the study was compromised by a lack of primary care input and by patient attrition, partly due to patient movement and also to low motivation in patients to adopt lifestyle change.^33^

Four physical fitness and weight management programmes were evaluated in five papers.^19^^,^^20^^,^^27^^,^^32^^,^^35^ A UK-based pre-post 10–12-week weight management and fitness programme was associated with a mean reduction of 1.3 kg (SD 2.73, range 12 kg gain to 9 kg loss) in weight and a 2.0 cm (SD 3.73, range 8 cm gain to 8 cm loss) reduction in mean waist circumference.^19^ Similar results were reported after 7 years of providing the programme; of 120 patients enrolled (excluding results from patients re-entering the programme), 63% lost weight, with 21 losing at least 5 kg. The total recorded mean weight loss across the 120 patients was 1.3 kg (range 12 kg gain to 11 kg loss). It is not known how well results were sustained in individuals over time. Male patients and those with learning disabilities responded better to participation in the programme than did women. However, there was a greater reported weight loss in women, possibly due to a higher baseline level of obesity in female residents.^20^ Addressing the gender discrepancy in physical activity uptake, Savage^32^ aimed to encourage women's participation through the delivery of a UK-based 12-week one-to-one programme covering physical assessment, education and physical activity. Six women provided data across a suite of psychological measures, which showed improved mood after participating in the sessions compared with before and increased motivation (with an associated increase in attendance) for the final four sessions. In the same organisation, Long^27^ evaluated a 30-min physical activity intervention (including staff training) in the UK, for women in low and medium secure units, incentivised by regular prompts, activities in break times and small monetary rewards. After 3 months all female patients were participating, and significant positive results included improved motivation and attendance as well as lower pulse rate (*P* < 0.01). Although these studies were small scale and lacking a control, the results indicate promising strategies for improving engagement with physical activity in both patients and staff. Feedback on an Australian exercise programme^35^ suggested that patients use the gym mainly to stay healthy and for enjoyment. Reported benefits included improvement in patient stress levels and self-care knowledge, as well as increased skill acquisition and social interaction. Negative feedback was from a small number of females who found physical activity difficult. There were no reported weight change outcomes in this paper, although 15.4% of respondents reported an increase in their fitness level.

Increased physical activity was the focus of one Australian case study that included two patients, assessed during their use of Wii Fit. Findings highlighted the importance of competition for the male patient and social interaction with staff for the female patient. These two factors increased their enjoyment of a potentially isolated activity. Both patients required initial encouragement from staff, owing to scepticism or perceived difficulty. Encouragement led to progressive use and enjoyment of the Wii Fit. Results for both patients were short-term weight reduction (one patient lost 1 kg after 5 weeks, at which point the patient was transferred to another unit; the other patient lost 3.4 kg after 8 weeks). The authors were keen not to emphasise weight loss as the main outcome in this study. This was because, while other activities could produce similar or greater levels of energy expenditure, the Wii Fit sessions also provided information about other health-related behaviours such as healthy eating.^34^

A multi-component (diet and physical activity) healthy lifestyle initiative was evaluated in New Zealand,^37^ motivated by the death of a young patient due to poor physical health. The initiative comprised two programmes, Programme A and Programme B. Programme A was embedded in ward routine and therefore compulsory. Evaluation suggested improved confidence and self-esteem for patients and staff. Programme B was not embedded and was available to only a sample of patients, which created some resentment. Implementing this programme highlighted challenges such as limited available space on the ward. The programme evolved as less authoritative and more patient-focused, particularly taking into account the rights of patients in terms of imposing restrictions.

As a result of study findings from a UK study, Long *et al*^25^ reported changes in ward or unit policy. These changes included limiting spending on, or access to, unhealthy foods in the tuck shop, controlling portion size, encouraging healthy eating options and restricting second helpings in canteens, as well as limiting takeaway orders.

### Staff support

Findings from included papers suggested that staff behaviours are a potential influence on patient lifestyle, with positive role modelling in eating behaviours^25^^,^^30^ and carrying out physical activities^34^ being areas that were identified for improvement. There was reported resistance to change,^37^ although eventually the changes became embedded into ward life. Facilitators to driving policy change included the efforts of ward champions^37^ and involving patients in ward policy discussions.^24^^,^^25^^,^^29^

### Organisational support

Support at the organisational level was required to ensure adequate facilities, staff resources and training for staff.^26^ Factors reported in successful initiatives included the provision of simple, practical information and messages,^36^ and raising the motivation of staff and patients on wards.^26^ Such changes could address resistance to change in both staff and patients. Thus, mental health secure units appear well placed to create an enthusiastic atmosphere^37^ compared with delivering weight management interventions within the community.

### Factors affecting uptake of behaviour change initiatives

From the papers included in this review, a number of factors were identified that appeared to motivate patients to engage with lifestyle change activities. These include interaction with staff and trainers, as well as a holistic^34^ and less authoritative approach^37^ that does not focus purely on weight.^34^ Activities that were enjoyed were shown to have positive effects on confidence and self-esteem,^27^^,^^32^^,^^37^ increase skills and knowledge^34^^,^^37^ and include social interaction.^35^^,^^37^ Patients might also value a competitive element to physical activities.^34^ However, they could be demotivated to participate owing to their mental health condition^24^^,^^27^ or because activities were perceived as too difficult.^34^^,^^36^ Resistance to change was a reported issue in both staff and patients.^36^^,^^37^

### Factors affecting implementation of behaviour change initiatives

A number of issues were evident in the literature that might affect implementation of change in mental health secure units. The tension between advocating patient autonomy and imposing control over access to preferred food was raised by authors.^21^^,^^24^^,^^28^^,^^30^^,^^36^^,^^37^ Tensions could be addressed by keeping patients involved in decision-making throughout the change process. The movement of patients in and out of units can affect outcome measurement when evaluating physical health initiatives.^19^^,^^20^^,^^33^^,^^34^ Maintenance of lifestyle change following discharge is also an issue for consideration^37^ which could be facilitated through referrals or links to community services on discharge.^35^

## Discussion

We carried out a review and synthesis of mixed method evidence relating to obesity in mental health secure units, to identify different aspects of the issue such as the extent of the problem, the types of intervention being tested and how feasible these might be in practice. We identified and critically appraised a total of 22 papers describing 21 studies of varied design. Findings indicate that an obesity problem exists in mental health secure units and that there is a need for more attention to monitoring physical health in these settings, in order to identify problems early and help prevent a number of obesity-related conditions.

The included studies were not able to show long-term effectiveness of particular interventions or components owing to the lack of comparator studies with long follow-up times. Given that effectiveness of interventions to improve BMI, weight and related outcomes have provided the basis for recent National Institute for Health and Care Excellence guidelines on obesity in the general population,^42^ the issues within mental health secure units are more about intervention implementation within this context. The findings of intervention studies included here thus provide an overview of promising directions for changing the obesogenic environment within secure units, while the included qualitative work identifies barriers to change, although currently the patient voice lacks representation.

The synthesis of evidence shows that reducing obesity in mental health secure units requires intervention that includes environmental, (facilities, space, design), educational (staff and patients) and service provision (assessing and monitoring physical health, intervention development, catering, physical activities) elements. Maintenance and motivation could be increased through staff champions, as well as by providing activities with realistic aims that are socially interactive and fun.

Long *et al*,^9^ in a review of healthy lifestyle interventions in secure units, supported the value of involving staff and patients in the endeavour to change the culture and environment within mental health secure units to facilitate an integrated approach to improving physical health.

Improving well-being in mental health secure units has been the aim of other work carried out by Public Health England, leading to recommendations in commissioning guidance for smoking cessation, where the opportunity to reduce smoking while patients are receiving NHS care was acknowledged.^43^ Smoking cessation and obesity reduction both address the 2017/2019 Commissioning for Quality and Innovation national indicator to reduce premature mortality in people with SMI.^44^ The secure unit setting provides a relatively consistent environment within which to intervene to promote physical health, although policy change in these settings needs to take account of the Mental Health Act 1983.^11^

The influence of lifestyle interventions in populations with serious mental health conditions outside secure unit settings have previously been reviewed, including smoking cessation,^45^ ways of improving nutrition,^7^ increasing physical activity,^46^^–^^48^ improving glycaemic control^49^ and preventing metabolic syndrome in schizophrenia.^50^ In one review of RCTs, although a small increase in physical activity rates was detected following intervention, no specific way of optimally improving physical activity levels was reported, and no effect on BMI, weight or mental health symptoms was identified, mainly owing to the heterogeneity of intervention types, settings and outcomes.^47^ In other reviews, increased physical activity had an effect on cognitive function^51^ and psychological well-being.^46^ Most intervention to preventing metabolic syndrome in schizophrenia tended to report some benefit, and monitoring physical health was identified as the key factor, although authors report that, methodologically, the evidence was poor.^50^

The existing literature thus shows that robust evidence is widely available for addressing obesity generally, and that RCTs with SMI populations outside secure units have been reviewed, although the evidence for effectiveness is less strong. This review shows that the evidence of effectiveness within secure units is less robust owing to sample sizes and study design, yet the contextual information is important and reflects the wider societal determinants of obesity. The Foresight Report^52^ uses a public health model to summarise the main influences on the rise of obesity prevalence generally, and emphasises the futility of tackling obesity using isolated activities at a purely individual level, given the interrelatedness of determinants and the role of organisational cultures. The culture within organisations, as well as determinants of health, would appear to be at least as influential for residents within secure units, where mental health conditions, medication and constraints within the environment can affect attempts to maintain physical health. As pointed out in previous work,^43^ it is important to aim towards the improved well-being for all people with mental health problems, and continuity of support can be offered within secure unit settings.

### Strengths and limitations

The evidence base around obesity in adult mental health secure units is limited and lacks robust studies compared with studies carried out in SMI or non-SMI populations within community settings. However, the available literature specific to secure units gives some indication of the change mechanisms that might influence specific obesogenic factors within units. With the exception of gender differences, there was scarce evidence relating to sub-groups such as ethnic minorities. However, the evidence does show that intervention and change require consideration of different needs and preferences to increase uptake and acceptance. There was a lack of patient voices within the included papers, although some studies included patient views in their evaluations of lifestyle change programmes.

## Future directions

The evidence reviewed here shows that a greater problem of obesity prevalence exists in mental health secure units than in the rest of the population. There are opportunities therefore to intervene while patients remain in care, in order to optimise levels of physical health and prevent undue obesity-related health problems. This is particularly pertinent in view of likely medication effects.

There are suggestions in the literature of how interventions might be carried out and of the barriers to implementing change. However, there is a need to establish the acceptability to patients, carers and staff of particular types of intervention. There is also a need for larger-scale evaluations with comparator designs and longer follow-up times. This will strengthen the evidence base for interventions that are feasible, acceptable and effective.
